# Ultra-broadband Asymmetric Light Transmission and Absorption Through The Use of Metal Free Multilayer Capped Dielectric Microsphere Resonator

**DOI:** 10.1038/s41598-017-15248-1

**Published:** 2017-11-06

**Authors:** Amir Ghobadi, Sina Abedini Dereshgi, Bayram Butun, Ekmel Ozbay

**Affiliations:** 1 NANOTAM-Nanotechnology Research Center, Bilkent University, 06800 Ankara, Turkey; 2 Department of Electrical and Electronics Engineering, Bilkent University, 06800 Ankara, Turkey; 3 Department of Physics, Bilkent University, 06800 Ankara, Turkey; 4 UNAM-Institute of Materials Science and Nanotechnology, Bilkent University, Ankara, Turkey

## Abstract

In this paper, we propose a simple design with an excellent performance to obtain high contrast in transmission asymmetry based on dielectric microspheres. Initially, we scrutinize the impact of the sphere radius on forward and backward transmissions. Afterward, by introducing a capping layer on top of the sphere, transmission response for the backward illuminated light will be blocked. In the next step, in order to replace the reflecting metal cap with a metal free absorbing design, we adopt a modeling approach based on the transfer matrix method (TMM) to explore an ideal material to achieve metal free perfect absorption in a multilayer configuration of material-insulator-material-insulator (MIMI). As a result of our investigations, it is found that Titanium Nitride (TiN) is an excellent alternative to replace metal in a MIMI multilayer stack. Setting this stack as the top capping coating, we obtain a high contrast between forward and backward light transmission where in an ultra-broadband range of 400 nm–1000 nm, forward transmission is above 0.85 while its backward response stays below 0.2. Moreover, due to the existence of multilayer stack, a high asymmetry is also observed for absorption profiles. This design has a relatively simple and large scale compatible fabrication route.

## Introduction

Asymmetric light transmission utilizing reciprocal electromagnetic (EM) systems is a burgeoning field of study on account of its potential applications in directional beam splitting^1,2^, multiplexing^3^, and optical interconnections^4^. An optical diode can be evaluated by considering the level of transmission contrast between forward and backward illuminations. This asymmetric response was initially attained through the utilization of a variety of configurations. Magneto-optical^5–7^, nonlinear^8–10^, and gyro anisotropic^11^ designs are examples of these devices. Contrary to the nonreciprocal transmission response obtained with these systems, metamaterials can offer asymmetric transmission that is reciprocal and fully obedient to Lorentz’s reciprocity theorem. Therefore, these metamaterials have been extensively exploited to provide transmission asymmetry for linear and circular polarizations. The use of double-sided nonsymmetrical grating structure^1–3,12,13^, chiral slabs^14–16^, split ring resonators^17,18^, and other hybrid architectures^19–21^ have been demonstrated as promising planar designs for this application. Particularly, if propagation asymmetry for a specific polarization is our interest, it can be realized using planar configurations that convert one polarization into the other. Photonic band gap structures are another category of designs that offer asymmetric response^22,23^. It was recently proved that a hyperbolic metal-dielectric multilayer sandwiched between two nonsymmetrical nanoresonant structures can propose a giant transmission contrast^24,25^. Employing accurately designed metasurfaces is another class of designs to achieve this property^26–28^. However, two important issues are raised taking the aforementioned ideas into consideration; 1) most of these works are implemented for infrared (IR), terahertz (THz), and gigahertz (GHz) regions that are far from visible light, and 2) they mainly provide this asymmetric response in a narrow frequency range.

In a very recent study, authors numerically demonstrated that a broadband unsymmetrical visible light transmission can be realized employing a tapered metallic grating structure^29^. The authors showed that forward transmission reaches to a value as high as 0.95 while this amount stays below 0.35 for backward incident light (a forward to backward transmission ratio of approx. 2.5) throughout a wavelength range of 550 nm–700 nm. However, similar to previous works, this design can also suffer from fabrication complexity since making a relatively thick tapered metal grating is a challenging task.

In this work, we propose and numerically demonstrate a simple yet high performance design architecture based on a dielectric microsphere resonators. We first analyze light propagation through the microsphere and the impact of its radius in the wavefront of the incident light. Afterward, taking the lensing advantage of spherical structure (with dimensions larger or comparable with the incoming light), the light transmission is blocked by introducing a capping layer. It is shown that this capping coating can efficiently prevent light propagation in the backward direction but does not affect its passage in the opposite direction. This capping layer is made of a metallic material that reflects light back into the cavity. However, there are two issues with this configuration that limit its efficiency. The first is stability against corrosion and oxidation under high temperature operation that is a possible condition for this design, considering the formation of highly focused hot spots at the top of the sphere (the position that capping layer is placed), and second is the reflection property of the metal which can lead to multiple reflections from the cap and this in turn can reduce the transmission contrast of the design. To tackle these deficiencies, we need to have an ultra-broadband light absorber to trap light inside the layer and prevent its reflection. On the other side, considering the curved surface of the sphere, the proposed design should be lithography free in order to be able to fabricate it. However, most of the present designs in the literature are composed of nanoresonant units which trap and confine the light in sub wavelength geometries. Recently, it was theoretically demonstrated that the use of a metal-insulator multilayer can provide ultra-broadband light absorption. In this configuration, the absorption edge is shifted toward longer wavelengths as the number of pairs is added up^30^. Since that work, several different metal-insulator configurations were utilized to prove the functionality of this design in the ultra-broadband absorption of the light^31–34^. The planar lithography free nature of these layers makes them an excellent choice for our capping coating. Taking this configuration into consideration, we propose the use of material-insulator-material-insulator (MIMI) multilayer configuration (instead of thick reflecting metallic layer) as the capping layer for the microspheres. To achieve this goal, by adopting a systematic modeling approach based on transfer matrix method (TMM), the ideal material for perfect light absorption is defined. Next, we compare the matching condition of a different class of materials with the ideal case. Our findings prove that titanium nitride (TiN) is an excellent choice for our desired frequency range. TiN is a refractory ceramic coating with a melting point of 2930 °C which makes it a stable coating for our application. Upon optimizing the geometries for the MIMI structure, an average absorption of 0.95 in a broad frequency range of 400 nm–1000 nm can be attained using this multilayer configuration. Taking this design as the capping layer, this time we evaluate the transmission asymmetry property of the capped microsphere structure. The results reveal that the aforementioned design configuration provides a relatively flat forward transmission as high as 0.85 while its backward response is suppressed below 0.2 in an ultra-broadband wavelength range from 400 nm to 1000 nm.

## Results and Discussion

**Figure 1:**
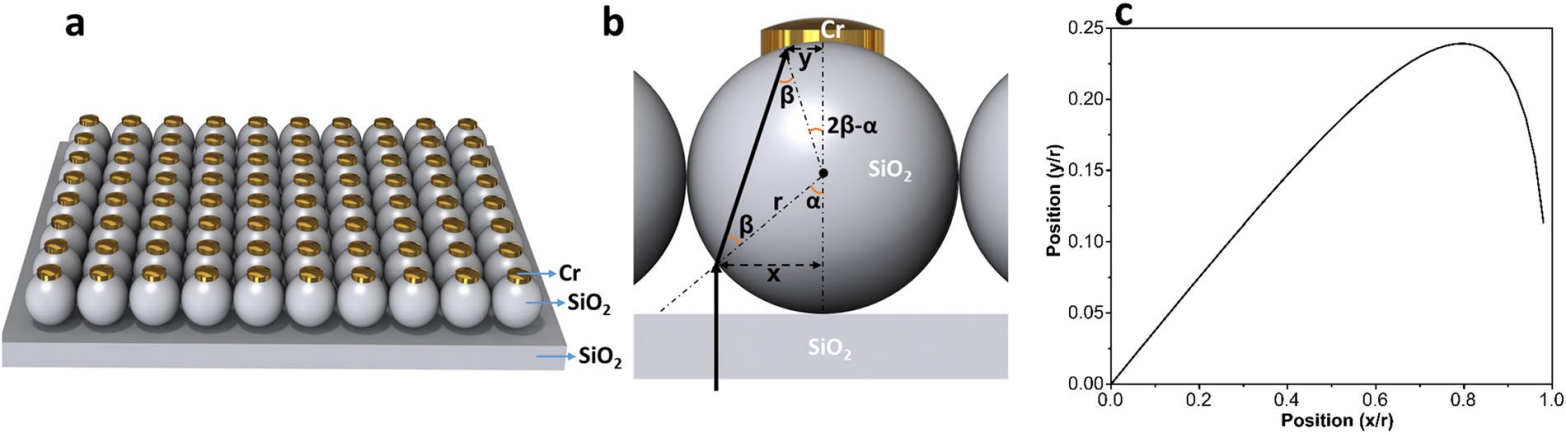
Illustrative schematic of the (**a**) proposed metal capped microsphere design, (**b**) ray propagation inside of microsphere, and (**c**) the light ray trace as the function of incidence position.

To begin with, we first study the light transmission through a metal capped dielectric microsphere. Figure 1(a) illustrates a schematic of the proposed architecture. The design is composed of a periodic arrangement of SiO_2_ microspheres capped with a Cr reflecting metal where the whole structure is fabricated on top of a SiO_2_ substrate. In the case that the microsphere dimensions are larger than (or comparable with) incident wavelength, as the first assumption, we can look at this structure as a ray optics problem. In other words, refraction mechanism (cusp catastrophe and caustics) is dominant instead of diffraction. It should be mentioned that this is assumed only to get an initial estimation about the wave propagation contour inside the sphere. Figure 1(b) depicts the ray path through the sphere. Applying Snell’s law in SiO_2_-air interfaces, we can estimate the light transmission route inside the microsphere using the following equation;

1 $$\frac{y}{r}=\,\sin \,(2{\sin }^{-1}(\frac{x}{nr})-{\sin }^{-1}(\frac{x}{r}))$$

Where the parameter n is the refractive index of the microsphere material that, in our case, is 1.45 for SiO_2_. The ray path profile is plotted in Fig. 1(c). As we can see from this figure, a normal incident light, in different lateral positions, will experience different deviations from its incidence line. As light goes away from the sphere center, the refracted light also gets larger distances from the central line. The maximum deviation is recorded at the incoming light position of *x* = 0.8*r* where the position of the light on the top metal cap is *y* = 0.24*r*. Therefore, assuming that ray optics estimation is valid for our geometries, it can be concluded that the incoming plane wave will be concentrated into a circle with a radius of 0.24*r*. This behavior has been reported from a ball lens and photonic nanojet^35–40^. To be able to provide a complete block of light transmission, the radius of the hat (or capping metal) is chosen as *r*_*hat*_ = 0.25*r* where the parameter *r* stands for the microsphere radius.

**Figure 2:**
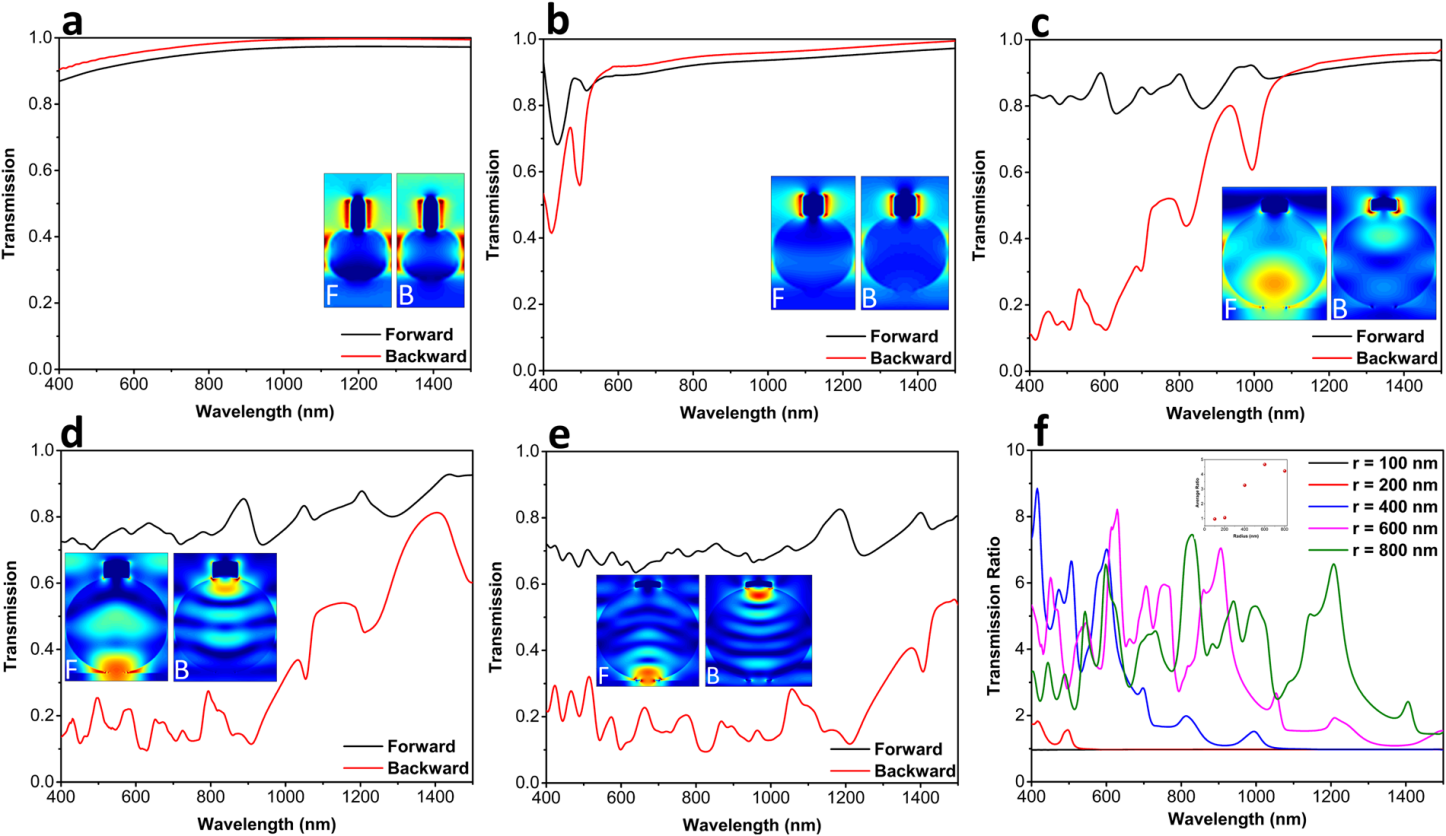
Forward and backward light transmission spectra for the different microsphere radii of (**a**) 100 nm, (**b**) 200 nm, (**c**) 400 nm, (**d**) 600 nm, and (**e**) 800 nm. The inset shows electric field distribution for two different incoming lights. Panel (f) reveals the forward to backward transmission ratios for different microsphere radii.

The impact of microsphere radius on the transmission of the light for backward and forward illumination is studied in the Fig. 2. In the inset of each graph, the electric field (E-field) distribution at the wavelength of *λ* = 800 nm is plotted. We carried out numerical simulations utilizing the commercial finite-difference time-domain (FDTD) software package (Lumerical FDTD Solutions)^41^. In these simulations, for the SiO_2_ and Cr layers, the Palik’s model is utilized^42^. As it can be deduced from Fig. 2(a,b), the transmission of the light for backward and forward incidence cases is almost the same for the small radii of *r* = 100 nm, 200 nm. This can be explained considering the size of the dielectric microsphere. In general, when a plane wave impinges on the surface of a particle, according to its dimension, it can experience two main types of scattering; 1) Rayleigh scattering and 2) Mie scattering. The first scattering mechanism is dominant if the radius of the particle is much smaller than that of the incident light wavelength ($kr\ll 1,$ where *k* is light wave number) which is handled with the well-known ray optics or cusp catastrophe and caustics. The second one, however, appears in large particles where rigorous Mie theory should be applied to study the wave-optical nature where photonic nanojets studies rely^39^. In the case of Rayleigh scattering, when $\,kr\ll 1$, the particle experiences a uniform E-field that is slowly oscillating in time and this incident E-field induces a time-varying Hertzian dipole moment in the sphere. Looking at the E-field profile of *r* = 100 nm case for forward and backward illuminations (as shown in the inset), we can clearly see the existence of a dipole moment in the middle plane of the sphere (light spots at the left and right positions of the sphere). Moreover, the mode profile is almost the same for both forward and backward incoming lights. This is due to the fact that the wavelength of the incident light is much longer than the particle dimension. Therefore, the asymmetric geometry of the sphere (where one side of the sphere is capped with a metal hat) cannot be identified with the incoming wave. This can be proven by taking the weak E-field light distribution inside the sphere into account. As this figure implies, light just passes the particle without diffusing into it. Moving toward lager spheres, the light penetration inside the microsphere is more visible. This can be clearly observed in the insets of Fig. 2(c–e) that correspond to the cases of *r* = 400 nm, 600 nm, 800 nm. For the microsphere structure with a radius of 400 nm, a distinct asymmetric response is found for smaller wavelengths. However, while moving toward larger wavelengths, the forward and backward profiles coincide with each other. This asymmetry is even more distinguishable for the cases of *r* ≥ 600 nm. As the ray optics dictates, when dimensions of the structure become larger than the light wavelength, a wave can penetrate inside the structure and its wavefront will take the same shape of the design surface. For the case of a microsphere, upon the illumination of the structure with a plane wave, light takes a spherical shape inside the sphere and gets focused on the other side of the sphere as a hot spot. These hot spots are clearly visible in two top and bottom sides of the sphere, as shown in Fig. 2(d,e). Dealing with Mie theory solutions^40^ falls beyond the focus of this study and FDTD simulations are sufficient. Putting a metal cap in one side of the sphere will block light transmission in one direction but will not affect it in the other one. Therefore, for any wavelength that is smaller than the sphere dimensions, employing this architecture yields an ultra-broadband asymmetric transmission response. Figure 2(f) depicts forward to backward transmission ratios for different cases. It is clearly shown in this figure that as the diameter of the microsphere gets larger, the bandwidth of this asymmetric response widens. To have a better qualitative comparison, we have restricted our desired wavelength band into a range of 400 nm–1000 nm and calculated the average transmission ratios for different microspheres cases. As illustrated in the inset of Fig. 2f, the highest average ratio belongs to the case of *r* = 600 nm, where an average transmission ratio of 4.7 can be attained using a metal capped microsphere design.

**Figure 3:**
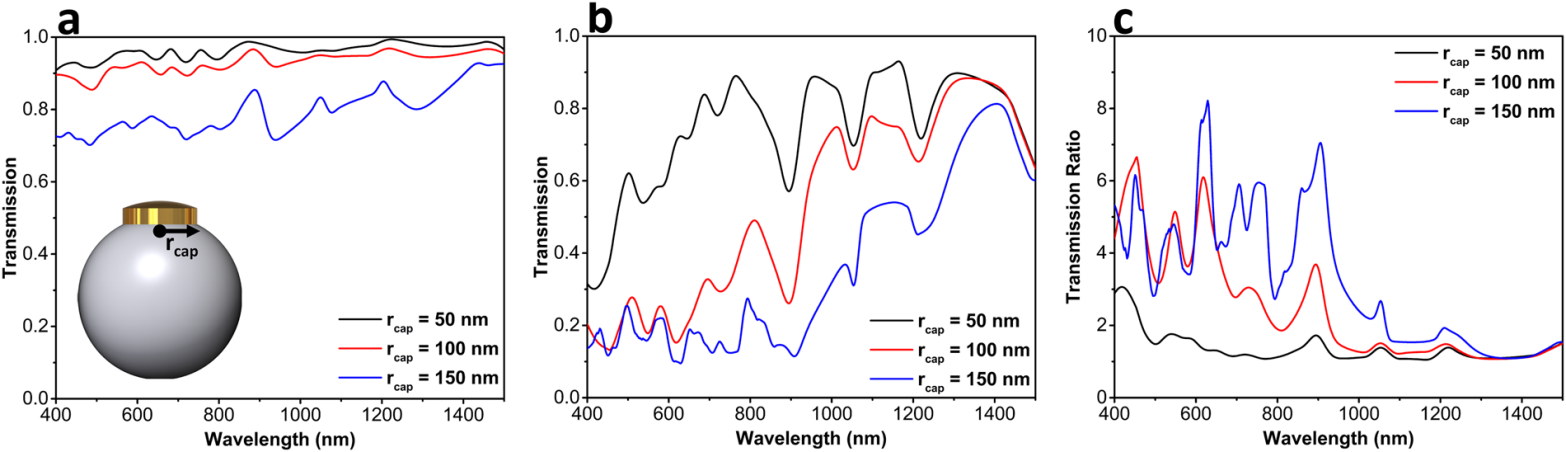
Transmission spectra for three different capping layer radii in (**a**) forward and (**b**) backward directions. Part (**c**) shows the transmission ratio for these three different designs.

Moreover, to understand the validity of our ray optics approximation, we swept the radius of the capping metal layer and explored forward and backward transmissions through the structure. Figure 3(a) plots the transmission values for forward illumination utilizing different cap radii of 50 nm, 100 nm, and 150 nm. Clearly, as the radius of metal capping layer reduces, more light can pass through the design. In the case of backward incident light, as shown in Fig. 3(b), 50 nm cap radius is not capable of blocking the incoming light. As a result, large transmission values are recorded for this case. For the case of 100 nm radius, the blocking property of the cap resembles that of 150 nm case, but it loses its response in longer wavelengths. This is expected considering the fact that light bending is more pronounced for shorter wavelengths where the dimension of the sphere is comparable or smaller than the incoming wavelength. Therefore, it is proven that our initial ray optics estimation is valid for the cap dimensions. This has also been evaluated by considering the transmission ratios for all three cases, as depicted in Fig. 3(c). The structure with *r*_*cap*_ = 150 nm retains its high transmission contrast in the wavelength range of 400 nm–1000 nm.

**Figure 4:**
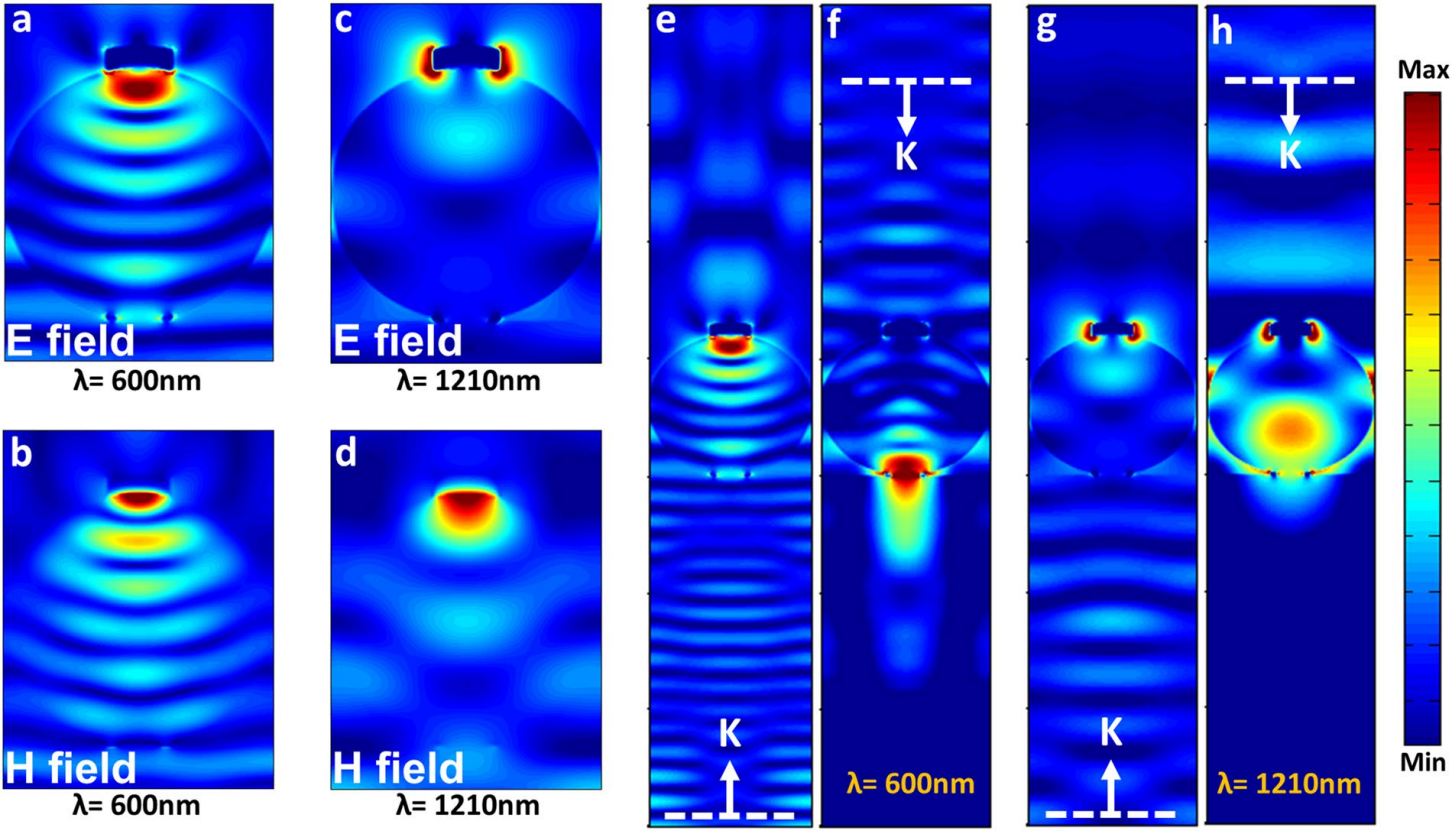
Electric and magnetic field distribution for two different wavelengths of (**a**,**b**) 600 nm and **(c**,**d**) 1210 nm, respectively. The light transmission profile for forward and backward incoming lights at wavelengths of (**e**,**f**) 600 nm and (**g**,**h**) 1210 nm.

**Figure 5:**
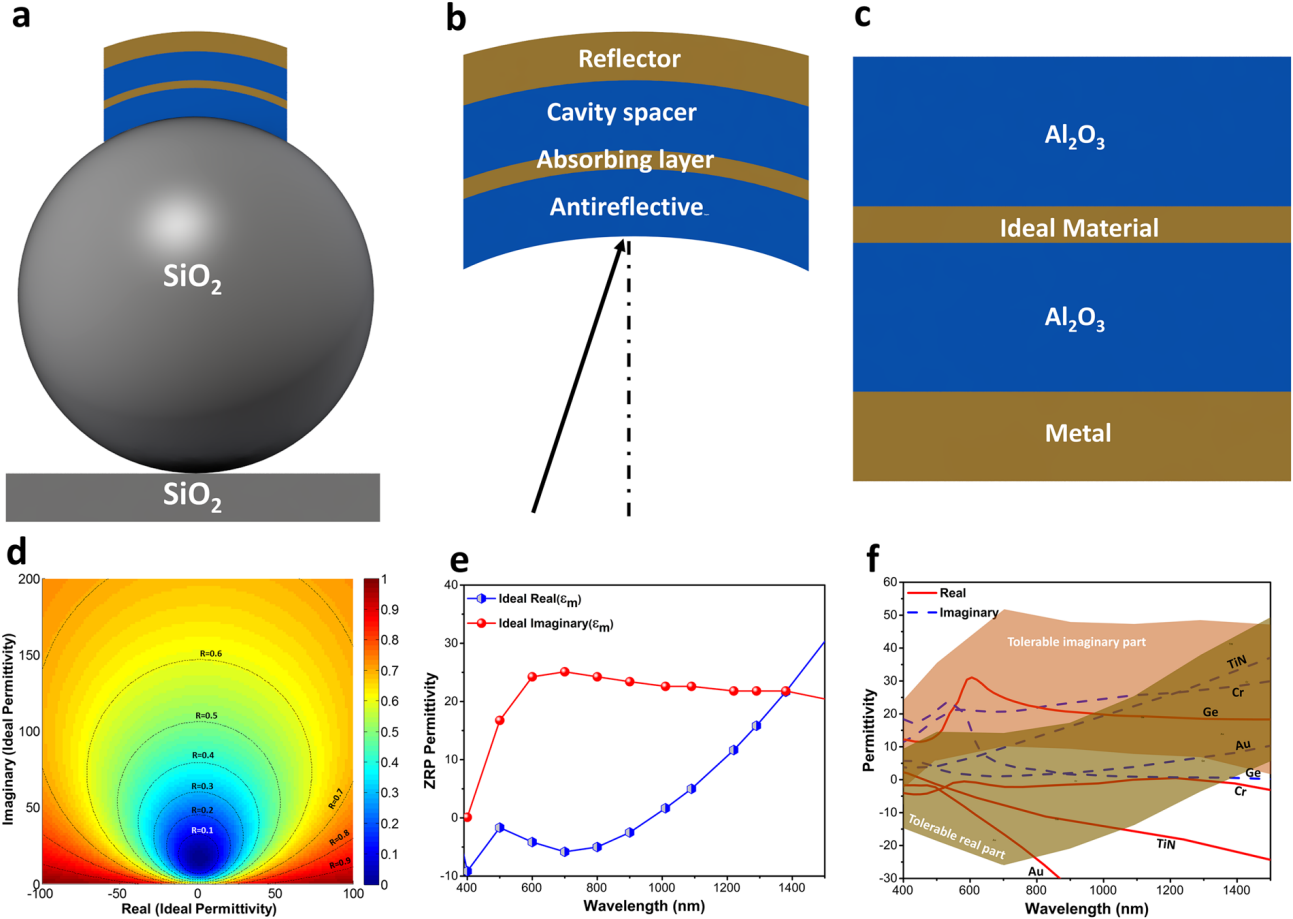
Schematic illustration of (**a**) proposed multilayer capped microsphere, (**b**) the role of different layers in light absorption and (**c**) its planar architecture for numerical investigation. Panels showing (**d**) reflection contour plots as a function of ideal permittivity, (**e**) the ZRP plot for real and imaginary parts of epsilon, and (**f**) the matching condition of different materials with the ideal model.

To gain more insight into the origin of light transmission asymmetry, both electric filed (E) and magnetic field (H) distributions at two different short and long wavelengths (*λ* = 600 nm and *λ* = 1210 nm) are plotted for the case of a structure with a microsphere radius of 600 nm. As Fig. 4(a) reveals, light is spherically focused into the top of microsphere and an interface pattern is created inside the sphere at *λ* = 600 nm. In the same wavelength, the magnetic field distribution is also confined in the sphere borders as it can be clearly understood from Fig. 4(b). This means that in shorter wavelengths, the total power is trapped in the particle and is focused into a hot spot point at the other side of the particle. However, in longer *λ* values, light wavefront radius curvature is larger than the metal cap radius, therefore, a capping layer cannot block the whole light and it diffracts from cap metal edges. This feature is shown in Fig. 4(c). In addition, the magnetic field distribution is no longer confined in sphere border and leaks out of the sphere region (as illustrated in Fig. 4(d)). A broader view can be accomplished looking at the farfield E-field distribution patterns for forward and backward incident lights. The mode profile for backward and forward light transmission at the wavelength of 600 nm is plotted in Fig. 4(e,f). As apparently depicted in these figures, while backward light is reflected back, the forward illumination gets a highly directional profile. However, the same directionality is not repeated for a longer incident wavelength (1210 nm) and a part of the incident light is concentrated out of the sphere, see Fig. 4(g,h). All of these findings prove the excellent transmission contrast in a metal capped microsphere architecture. This design performs as a mirror for backward light illumination and shows high transmissivity for forward incoming wave. However, as already mentioned, the drawback with this design is the fact that the portion of the light that reflects back from the metal cap can experience multiple reflections in which it will finally transmit to other side. This in turn will diminish the capability of the design to provide high contrast between two different incident directions. The other drawback with this design is the use of metallic material as the capping layer. Considering the formation of light hot spot in the position of capping layer, this metal layer can gain high temperatures. This consequently reduces its durability due to oxidation and corrosion issues. Therefore, an ultimate design should be metal free and should have the ability to trap and absorb light rather than reflecting it back into the design. Moreover, this design should satisfy fabrication compatibility factor as well. However, essentially, to attain light absorption, we need to have sub wavelength geometries in our design. These sub wavelength unit resonators are called plasmonic designs. One of the most commonly used ideas to achieve a high absorption response is to employ a metal-insulator-metal (MIM) cavity. In this design architecture, the bottom metal layer is an optically thick reflecting coating and top metal layer is a periodic arrangement of nanostructure resonator. The introduction of the bottom metal layer will reflect back the light into the cavity and overall light absorption will be enhanced compared to that of a single nanostructured plasmonic layer. However, patterned top layer design requires electron beam lithography (EBL) which is not only large-scale incompatible, but also it cannot be applied to our structure where the substrate is a curved sphere (top of the microsphere where the capping coating is introduced). Therefore, these common designs cannot be good choices for our architecture. In a recent study, it was theoretically demonstrated that the use of metal-insulator pair, where the metal layer thickness is ultrathin, can offer an ultra-broadband absorption response. Unlike MIM design, these {MI}_N_ multilayer stacks do not need any lithography process. Thus, it is a large scale compatible method to fabricate ultra-broadband perfect absorbers. Several different metal-insulator pairs have been utilized to get near unity broadband absorption from the structure. Cr-SiO_2_^32^, Mo-SiO_2_^32^, W-Al_2_O_3_^31^ are examples of the studied materials. Throughout these studies, it was found that the best absorption property can be acquired using a Cr-SiO_2_ pair combination in a MIMI architecture. Such a structure can retain absorption above 90 percent throughout a broad wavelength range of 400 nm–1400 nm. Nevertheless, to satisfy the durability issues, a more efficient design can be implemented with a metal free multilayer stack. To find a suitable material to replace with metal, we first need to know about the ideal material to get near unity absorption. For this aim, we adopted a systematic methodology based on transfer matrix method (TMM) to model the design. In this method, we suppose the MIMI structure is bounded with a material of ε_A_ which is the air in our case. For TM polarization, considering the H_y_ as:

2 $${{\rm{H}}}_{{\rm{y}}}(z)=\{\begin{array}{l}{{\rm{A}}}_{{\rm{i}}}{{\rm{e}}}^{{{\rm{ik}}}_{{\rm{A}}}{\rm{z}}}{+A}_{{\rm{r}}}{{\rm{e}}}^{{-\mathrm{ik}}_{{\rm{A}}}{\rm{z}}},\,\,\,z < 0\\ {{\rm{D}}}_{{\rm{11}}}{{\rm{e}}}^{{{\rm{ik}}}_{{\rm{I}}}{\rm{z}}}{+D}_{{\rm{12}}}{{\rm{e}}}^{{-\mathrm{ik}}_{{\rm{I}}}{\rm{z}}},\,\,\,\,{0 < z < D}_{{\rm{I}}}\\ {{\rm{M}}}_{{\rm{11}}}{{\rm{e}}}^{{{\rm{ik}}}_{{\rm{M}}}{(z-D}_{{\rm{I}}})}{+M}_{{\rm{12}}}{{\rm{e}}}^{{-\mathrm{ik}}_{{\rm{M}}}{(z-D}_{{\rm{I}}})},\,\,{{\rm{D}}}_{{\rm{I}}}{ < z < D}_{{\rm{I}}}{+D}_{{\rm{M}}}\\ {{\rm{D}}}_{{\rm{21}}}{{\rm{e}}}^{{{\rm{ik}}}_{{\rm{I}}}{[z-(D}_{{\rm{I}}}{+D}_{{\rm{M}}})]}{+D}_{{\rm{22}}}{{\rm{e}}}^{{-\mathrm{ik}}_{{\rm{I}}}{[z-(D}_{{\rm{I}}}{+D}_{{\rm{M}}})]},\,\,\,{{\rm{D}}}_{{\rm{I}}}{+D}_{{\rm{M}}}{ < z < \mathrm{2D}}_{{\rm{I}}}{+D}_{{\rm{M}}}\\ {{\rm{M}}}_{{\rm{21}}}{{\rm{e}}}^{{{\rm{ik}}}_{{\rm{M}}}{[z-(\mathrm{2D}}_{{\rm{I}}}{+D}_{{\rm{R}}})]}{+M}_{{\rm{22}}}{{\rm{e}}}^{{-\mathrm{ik}}_{{\rm{M}}}{[z-(\mathrm{2D}}_{{\rm{I}}}{+D}_{{\rm{R}}})]},\,\,\,{{\rm{2D}}}_{{\rm{I}}}{+D}_{{\rm{M}}}{ < z < \mathrm{2D}}_{{\rm{I}}}{+D}_{{\rm{M}}}{+D}_{{\rm{R}}}\\ {{\rm{A}}}_{{\rm{t}}}{{\rm{e}}}^{{{\rm{ik}}}_{{\rm{A}}}{[z-(\mathrm{2D}}_{{\rm{I}}}{+D}_{{\rm{M}}}{+D}_{{\rm{R}}})]},\,\,{z > (\mathrm{2D}}_{{\rm{I}}}{+D}_{{\rm{M}}}{+D}_{{\rm{R}}})\end{array}\}$$

and applying appropriate boundary conditions, reflection of the incident light from the structure can be obtained using ${\rm{R}}={|\frac{{\rm{F}}12}{{\rm{F}}11}|}^{2}$. Here,$$F=[\begin{array}{c}{F}_{11}\\ {F}_{12}\end{array}]={a}_{1}^{-1}{d}_{1}{d}_{2}^{-1}{m}_{1}{m}_{2}^{-1}{d}_{1}{d}_{2}^{-1}{a}_{2}$$ where:

3a $${a}_{1}=[\begin{array}{cc}1 & 1\\ i{k}_{A}/{\varepsilon }_{A} & -i{k}_{A}/{\varepsilon }_{A}\end{array}],\,\,{a}_{2}=[\begin{array}{c}1\\ i{k}_{A}/{\varepsilon }_{A}\end{array}],$$

3b $${d}_{1}=[\begin{array}{cc}1 & 1\\ i{k}_{I}/{\varepsilon }_{I} & -i{k}_{I}/{\varepsilon }_{I}\end{array}],\,\,{d}_{2}=[\begin{array}{cc}{e}^{i{k}_{D}{D}_{I}} & {e}^{-i{k}_{I}{D}_{I}}\\ i{k}_{I}{e}^{i{k}_{I}{D}_{I}}/{\varepsilon }_{I} & -i{k}_{I}{e}^{-i{k}_{I}{D}_{I}}/{\varepsilon }_{I}\end{array}],$$

3c $${m}_{1}=[\begin{array}{cc}1 & 1\\ i{k}_{M}/{\varepsilon }_{M} & -i{k}_{M}/{\varepsilon }_{M}\end{array}],\,\,{m}_{2}=[\begin{array}{cc}{e}^{i{k}_{M}{D}_{M}} & {e}^{-i{k}_{M}{D}_{M}}\\ i{k}_{M}{e}^{i{k}_{M}{D}_{M}}/{\varepsilon }_{M} & -i{k}_{M}{e}^{-i{k}_{M}{D}_{M}}/{\varepsilon }_{M}\end{array}],$$

and ${k}_{i=(A,I,M)}=\sqrt{{\varepsilon }_{i}{\omega }^{2}/{c}^{2}-{k}_{x}^{2}}$ where “c” is the speed of light. Moreover, D_I_, D_M_ and D_R_ are the thicknesses of the dielectric, middle material and reflector layers, respectively. Figure 5(a) depicts the proposed structure of a multilayer capped microsphere design. As Fig. 5(b) illustrates this MIMI structure has four distinct layers. As mentioned earlier, the first layer is acting as a broad mirror that reflects light back into the cavity. Then, an insulator spacer separates the reflecting layer from middle ultrathin metal coating. Finally, the last layer performs as an ultra-broadband antireflective coating to provide impedance matching between air and underlying MIM cavity. To find out the ideal material for our multilayer stack design, we utilize the proposed MIMI stack as shown in Fig. 5(c). First, the insulator layer thickness is fixed at 80 nm and is chosen to be Al_2_O_3_ (alumina). For the alumina layer, the Palik model is employed^42^. Then, the ideal material for a 10 nm thick middle material is investigated. It should be noted that the bottom metal layer is chosen as Cr, which is a good mirror throughout our desired frequency range. Figure 5(d) shows the contour plot for the reflection values of the structure as a function of real and imaginary parts of the ideal middle material. The reflection contour plot consists of centric circles (or semi-circles) where the radii of these circles get larger as we move to larger reflection values. Considering the fact that the bottom reflector layer is opaque, the ideal permittivity values belong to the case in which the reflection is zero (corresponding to a near unity absorption). We have extracted zero reflection points (ZRPs) for both real and imaginary parts of epsilon and plotted them in Fig. 5(e). This panel illustrates that the imaginary part of the ideal material has small values at lower λs and after an abrupt increase stays constant at an amplitude of approximately 25. On the other side, the real part stays at small negative values in lower wavelengths and gets an exponential increase toward positive values in wavelengths longer than 1000 nm. This is actually the main reason that the absorption bandwidths of metals are limited; the real part of permittivity values for a metal gets large negative values as we move toward longer wavelengths. Taking *R* = 0.1 circle (which corresponds to an absorption above 0.9) as our threshold for perfect light absorber definition, a tolerable region for real and imaginary parts of ideal material is highlighted at Fig. 5(f). Thus, if a material permittivity (both real and imaginary parts) stays inside this region, the MIMI stack would ensure absorption above 0.9. To examine how well different materials are matched into this ideal model, we have evaluated the permittivity data for 4 different materials; 1) Au (as a Nobel metal), 2) Cr (as a refractory metal), 3) Ge (as a small band gap semiconductor) and TiN (as a ceramic material). The optical data for Au, Cr, Ge and TiN layers have been all acquired from Palik’s handbook^42^. All of these materials have the minimum requirement of having absorption coefficient in large λ values but not all of them match to the ideal model. Among all, Au has the worst matching properties in which a real part of its permittivity leaves the highlighted region at a wavelength near 800 nm and its imaginary part is almost entirely out of the region. The other metal, which is Cr, has the best matching where both real and imaginary parts stay inside the region up to 1350 nm. This has been experimentally found that the use of Cr provides the widest absorption bandwidth where absorption above 0.9 was recorded from 400 nm to 1400 nm^32^. However, our initial goal was to replace this metal with a nonmetal stable material. Ge, as a semiconductor with a long absorption edge, has been also inserted in this panel. The findings reveal that the real part of permittivity for Ge stays out of the region for *λ* < 1000 nm, and its imaginary part is entirely out of tolerable borders. Unlike Ge, the other non-metal material that is TiN has excellent matching for both parts in a wide wavelength range covering from 400 nm to 1100 nm. Therefore, TiN is a promising material for replacing metal layer in a MIMI configuration.

**Figure 6:**
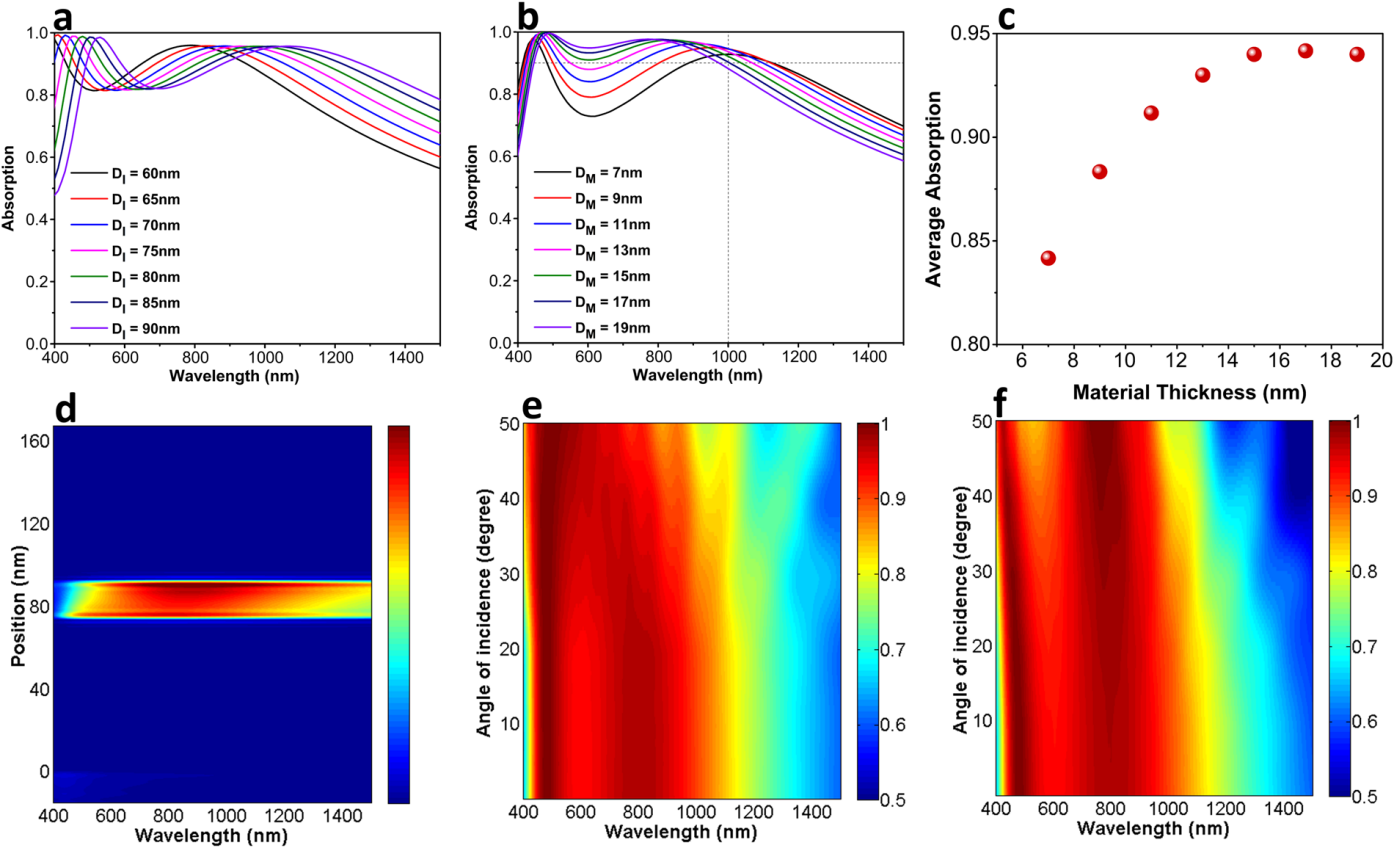
Absorption spectra for different (**a**) insulator thicknesses, (**b**) material thicknesses, (**c**) calculated average absorption values, (**d**) absorbed power distribution in different parts of the MIMI cavity, and oblique angle response for (**e**) TM and (**f**) TE incidence light polarizations.

Upon finding TiN as a proper choice for multilayer stack absorber, a study should be conducted to define the optimal geometries for the highest absorption capability. In the following study, the bottom reflecting layer thickness is taken as 100 nm to block the light transmission (a transmission below 1% was recorded throughout the whole frequency range of 400 nm–1500 nm). The thicknesses of insulator layer (D_I_) and middle TiN layer (D_M_) are swept to find the optimal response. Initially, the insulator layer thickness is tuned to get the best choice, see Fig. 6(a). Taking our desired operation range of 400 nm–1000 nm into consideration, the insulator layer thickness is chosen in a way that a relatively high absorption can be attained in lower edge of the range while the upper edge is extended beyond 1000 nm. To meet these requirements, the D_I_ value is assumed to be 75 nm. Fixing this value for the alumina layer, as depicted in Fig. 6(b), the impact of the middle TiN thickness is studied. As it can be clearly observed, moving toward thicker layers, the absorption spectra is improved in lower wavelengths but its upper edge also experiences a gradual blue shift and therefore, the absorption bandwidth is reduced. To have a good measure to compare these spectra, we calculated the average absorption of different cases (in 400 nm–1000 nm range) showing the flatness of the absorption response. Figure 6(c) reveals that the highest value belongs to D_M_ = 17 nm, where an average absorption as high as 0.94 can be achieved using the proposed geometries. To elucidate the role of different layers in a light absorption response of the MIMI multilayer, we have plotted the absorbed power as a function of incidence light wavelength through the whole stack (Fig. 6(d)). As it can be obviously explored from this graph, the absorbed power is mainly concentrated in the middle TiN structure which is an expected result taking the high extinction coefficient of this material into account. In fact, in this architecture, light is trapped inside the bottom MIM cavity until it is completely absorbed by the middle layer. Moreover, as we discussed in the previous section, the incoming light hits the capping layer with a specific angle. Therefore, the angle response of the system is of great importance. Figure 6(e,f) depicts the absorption contour plots for the transverse magnetic (TM) and transverse electric (TE) polarizations. As this panel explains, the absorption profile of the system keeps its high values for oblique incidence angles acceptably as high as θ = 50°. In the case of TM polarization, the absorption bandwidth would only reduce in a small amount but this reduction is more abrupt for TE polarized incidence light. However, for both cases, the absorption amount retains above 0.85.

**Figure 7:**
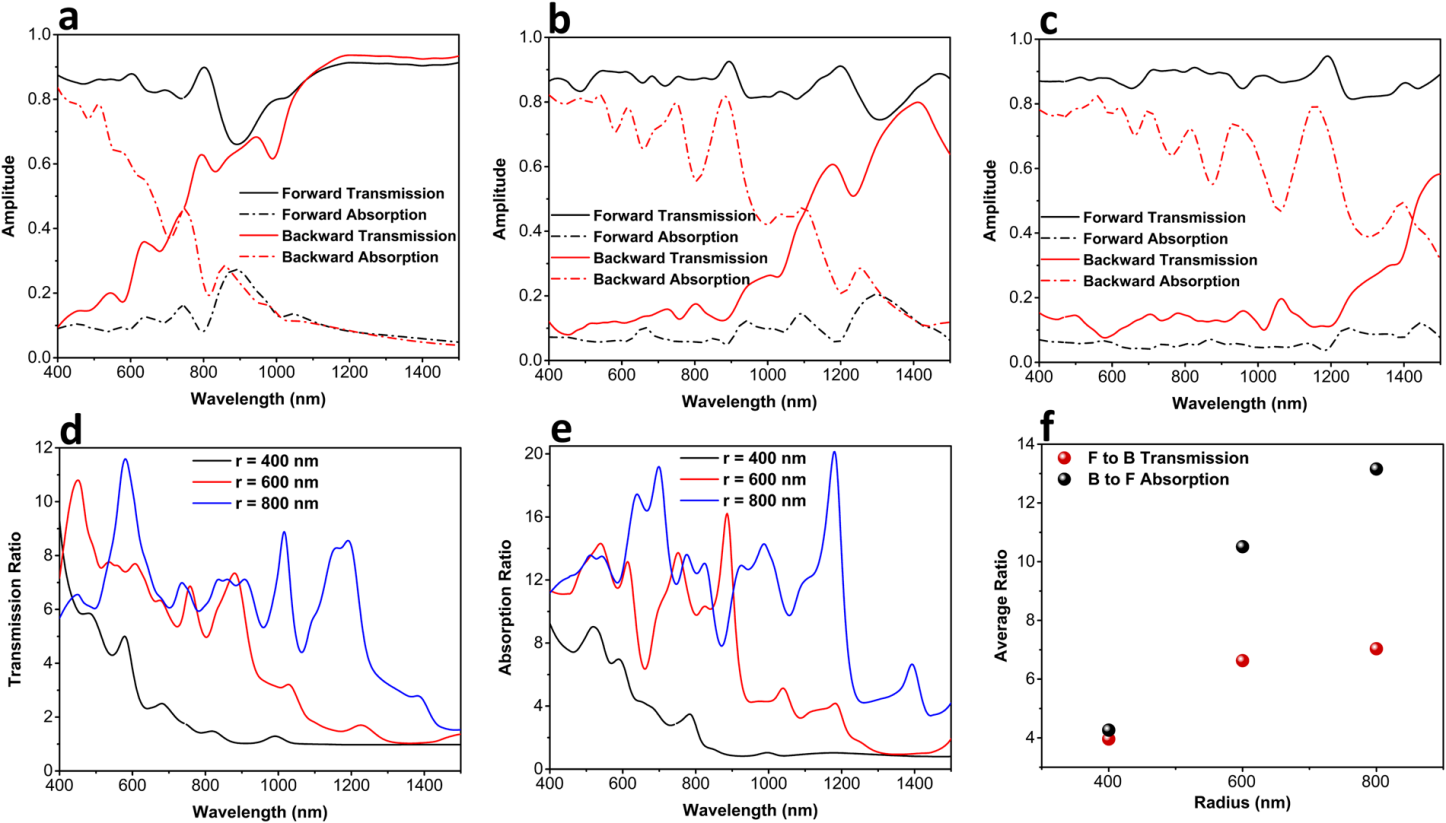
Transmission and absorption spectra for the different microsphere radii of (**a**) 400 nm, (**b**) 600 nm, and (**c**) 800 nm. Corresponded (**d**) transmission and (**e**) absorption ratios and (**f**) calculated average values for three different architectures.

**Figure 8:**
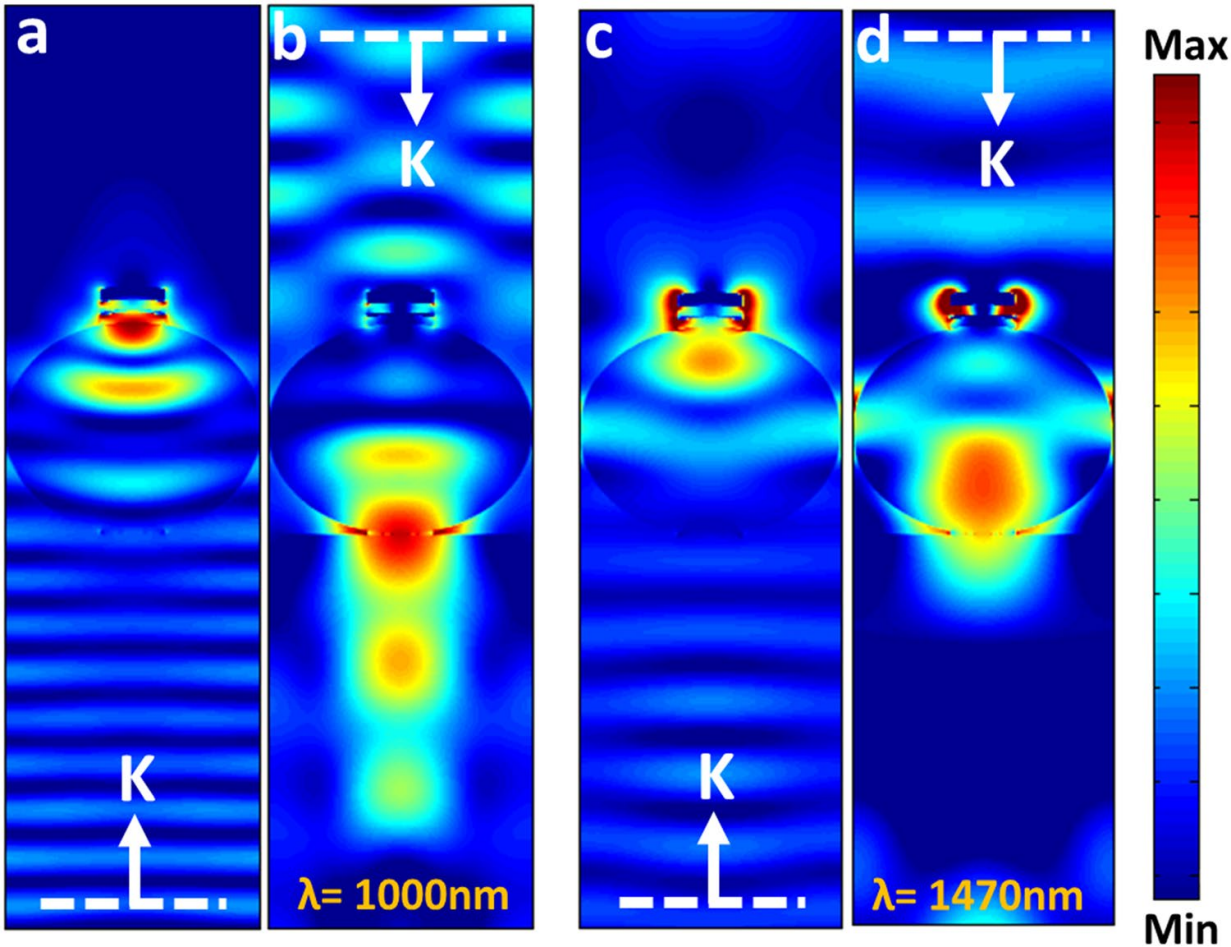
The light transmission profile for forward and backward incoming lights at wavelengths of (**a**,**b**) 1000 nm, (**c**,**d**) 1470 nm.

Putting the optimal MIMI design as the capping layer on top of microsphere, this time we have evaluated the asymmetric transmission capability of this system. Figure 7(a–c) plots the forward and backward transmission response for three different radii of *r* = 400 nm, 600 nm and 800 nm Unlike the previous case, where we used metal Cr only rather than a MIMI multilayer structure, this structure can also provide asymmetric absorption for two different directions of the light. Similar to the results presented at Fig. 2, as the radius of the sphere increases, the operation band of the design gets wider toward longer wavelengths. For the microsphere radius larger than 600 nm, a relatively high contrast is recorded between backward and forward illumination. This geometry provides a barely flat transmission with a value above 0.8 for forward illuminated light while backward transmission is kept below 0.2. For *r* = 600 nm, this condition is retained from 400 nm to around 920 nm while *r* = 800 nm can sustain its asymmetric property up to 1280 nm. This result can be also be observed in Fig. 7(d,e) where the ratios for forward to backward transmission and backward to forward absorption are plotted, respectively. A more illustrative comparison can be obtained by calculating the average transmission and absorption ratios for three different cases shown in Fig. 7(f). As we can see, for this configuration, the design with an 800 nm radius microsphere shows a transmission ratio of approximately 7.1, which is higher compared to that of the 600 nm one. Moreover, this value is higher than that of the metal capped configuration (Fig. 2(f)), in which the ratio of asymmetry was 4.7. This is mainly due to existence of a multilayer stack at the top of the sphere. Different from a bare metal reflector that reflects light back into the sphere, the MIMI cavity traps the light inside itself and avoids multiple reflections. Consequently, most of the backward light would be blocked and a flatter response can be obtained. Moreover, the design can also impose asymmetric light absorption where the backward to forward absorption ratio can reach to an amount of 13.2 for the case of *r* = 800 nm. These findings can be verified by simply looking at the electric field profile passing across the structure. Figure 8(a,b) display the electric field distribution for the backward and forward incident lights at *λ* = 1000 nm. As can be seen in this figure, the backward illumination is trapped inside the MIMI cap and is entirely blocked from propagation into the other side. On the other hand, the forward illumination has been bended toward the bottom corner of the sphere and has acquired a directional beam shape. Another prominent feature in these contour plots is the fact that the plane wave keeps its planar wavefront upon impinging the structure from backside. This feature cannot be observed in the case of metal capped microsphere architecture (see Fig. 4e). This again proves the ability of the MIMI structure in the absorption of the incoming light in a way that the reflection from the capped microsphere is almost zero and no interruption in the plane wave profile is observed. The same feature can also be recognized for the longer wavelength as shown in Fig. 8(c). However, as we already explained in Fig. 4, for larger values of λ the light curvature is larger than that of cap width and, therefore, the structure cannot efficiently block the light transmission.

The above results have been obtained for an ideal TiN layer model. However, it has been proved that the permittivity data of TiN layer significantly changes under different deposition conditions^43^. To evaluate the functionality of our design, we utilized these practical data for the TiN coating. The deposition conditions have been classified as 7 different samples as explained in the supplementary information. Similar to above mentioned optimization strategy, we first sweep the insulator layer thickness. Later the optimized planar configuration is attained with tuning the TiN layer thickness. Finally, the obtained optimized MIMI configuration is set as the capping layer for the microsphere (with the radius of 800 nm). As shown in Fig. S1(a–c), almost for all 7 different cases, a high contrast transmission and absorption asymmetry can be realized. This is mainly due to MIMI optical characteristic where a wide deviation for the ideal permittivity values can be tolerated. This fact can be seen in Fig. 5f. Finally, fabrication of this design can be also done with a feasible route. First, the SiO_2_ microspheres are coated on the substrate using a self-assembly or other methods which have been widely explored in the literature^44–48^. Afterward, this periodic arrangement of the spheres is coated with a photoresist and selectively etched with a wet or dry etching process in a way that the top of the spheres appeared. Then, using sputtering technique, TiN and Al_2_O_3_ layers are alternatively deposited on the sample. Finally, employing an ultra-sonication assisted lift off process the desired design can be made.

## Conclusion

In summary, in this study, we have developed an elegant design configuration to obtain high asymmetry in light transmission for different anti-parallel directions. The proposed design is made up of a periodic arrangement of capped microspheres. The capping layer is composed of a MIMI configuration to ensure light absorption in a wide frequency range. It was found that the optimal geometries for a planar multilayer stack offer a flat absorption above 0.9 across a range covering from 400 nm to 1000 nm. Employing this stack on top of a *r* = 800 nm SiO_2_ sphere provides high contrast light transmission asymmetry. It was shown that a relative flat transmission with a value around 0.85 can be obtained for the forward direction, while this value remains below 0.2 for backward incidence light. Moreover, this design has a relatively feasible fabrication possibility and as explained in the final section, its fabrication can be lithography free, which makes it large scale compatible.

## Electronic supplementary material

Supplementary Information
