# Supplementary material for: Ultra-broadband Asymmetric Light Transmission and Absorption Through The Use of Metal Free Multilayer Capped Dielectric Microsphere Resonator
Source: Sci Rep. 2017 Nov 6;7:14538. doi: 10.1038/s41598-017-15248-1 (PMC5674040; doi:10.1038/s41598-017-15248-1)
Supplement: Supplementary file 1 — Supplementary Information [file 41598_2017_15248_MOESM1_ESM.docx]

Supporting Information

subject areas: ASYMMETRIC LIGHT TRANSMISSION, oPTICAL DIODE, MICRO/NANO SPHERES, METAL INSULATOR MULTILAYER, ABSORBER

Correspondence and requests for materials should be addressed to Prof. Dr. Ekmel Ozbay

(E-mail: ozbay@bilkent.edu.tr)

**Ultra-broadband Asymmetric Light Transmission and Absorption Through The Use of Metal Free Multilayer Capped Dielectric Microsphere Resonator**

Amir Ghobadi**^1, 2, *^**, Sina Abedini Dereshgi**^1, 2^**, Bayram Butun**^1^**, Ekmel Ozbay**^1, 2, 3, 4, *^**

^1^ NANOTAM-Nanotechnology Research Center, Bilkent University, 06800 Ankara, Turkey

^2^ Department of Electrical and Electronics Engineering, Bilkent University, 06800 Ankara, Turkey

^3^ Department of Physics, Bilkent University, 06800 Ankara, Turkey

^4^ UNAM-Institute of Materials Science and Nanotechnology, Bilkent University, Ankara, Turkey

* Corresponding authors: [amir@ee.bilkent.edu.tr](mailto:amir@ee.bilkent.edu.tr) , [ozbay@bilkent.edu.tr](mailto:ozbay@bilkent.edu.tr)

In the previous study, the authors have demosntrated that the permittivity values of TiN layer can be tuned using modification of growth conditions such as gas flow ratios, annealing temperature, and the substrate type. We will use permittivity data of 7 different growth conditions to evaluate the functionality of our optical diode design.To address the impact of different deposition conditions of TiN on the performance of the design, we have classified them as follows:

*Different Ar:N_2_ flow ratios;*

Sample. 1: Ar:N_2_ = 4:6

Sample. 2: Ar:N_2_ = 2:8

Sample. 3: Ar:N_2_ = 0:10

*Different annealing tempratures;*

Sample. 4: Annealed at 500°c

Sample. 5: Annealed at 300°c

*Different substrates;*

Sample. 6: Glass

Sample. 7: c-Saphire

Using these different practical data, we have repeated our simulations to make sure that the transmission and absorption asymmetry of the design is hold under different TiN growth conditions. For this aim, we first optimized the result for a planar MIMI design and later the optimum layer is utilized as the capping layer to evaluate the asymmetry level of the design. Fig. S1(a-c) depicts the simulation results for all of these 7 different options. Similar to what we have done in the paper, we first conducted a sweep on the insulator layer thickness. Later the optimized results for the broadest light perfect absorption is attained by tuning the metal layer thickness. As shown in the below figure, for different samples, the optimized design dimensions are different. The absorption for the sample. 1 stays above 0.9 in a range of 400 nm- 1270 nm for the optimized dimensions of 65 nm-25 nm-65 nm (D_I_-D_M_-D_I_). However, the optimized configuration for the sample. 2 is found to be 65 nm-20 nm-65 nm. The essential conclusion from these graphs is the fact that almost all the designs can provide light perfect absorption in our desired wavelength range of 400 nm- 1000 nm. Therefore, it is expected that all of these different TiN layers can ensure assymetric light absorption and transmission. This has been proved in the panel (c) for different samples. As it can be seen from this figure, the assymetric response of the multilayer capped microspheres can be obtained from all the different cases. As already meantioned, this is due to design flexibility and high level of toleration in a MIMI configuration.


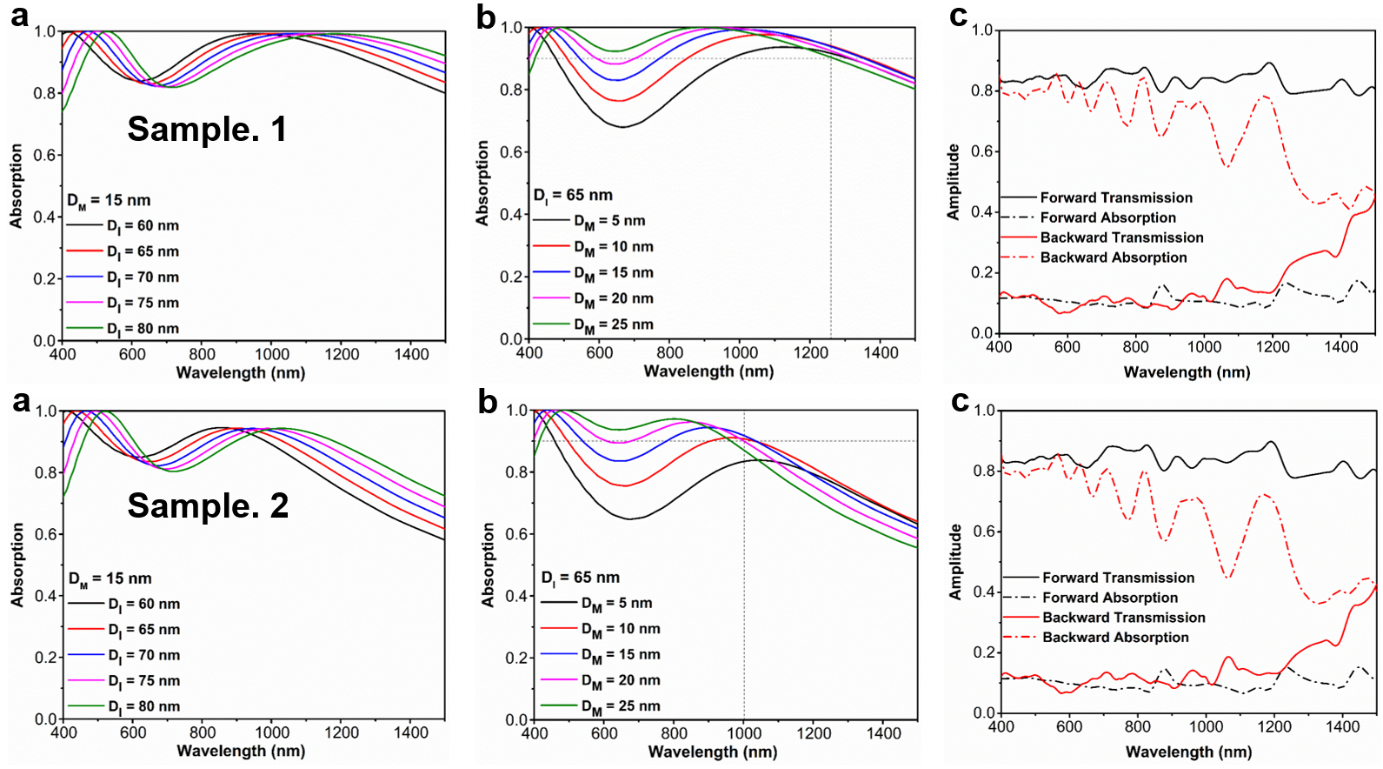

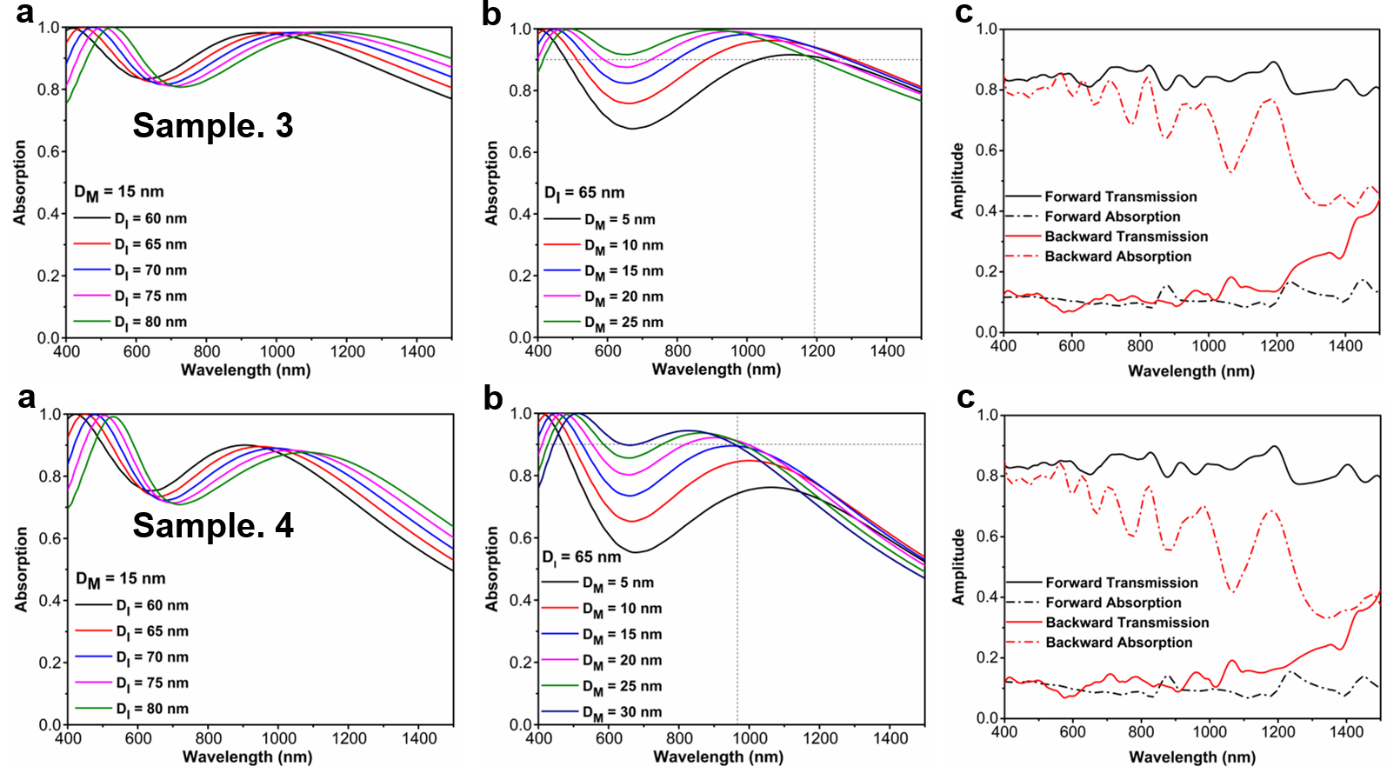

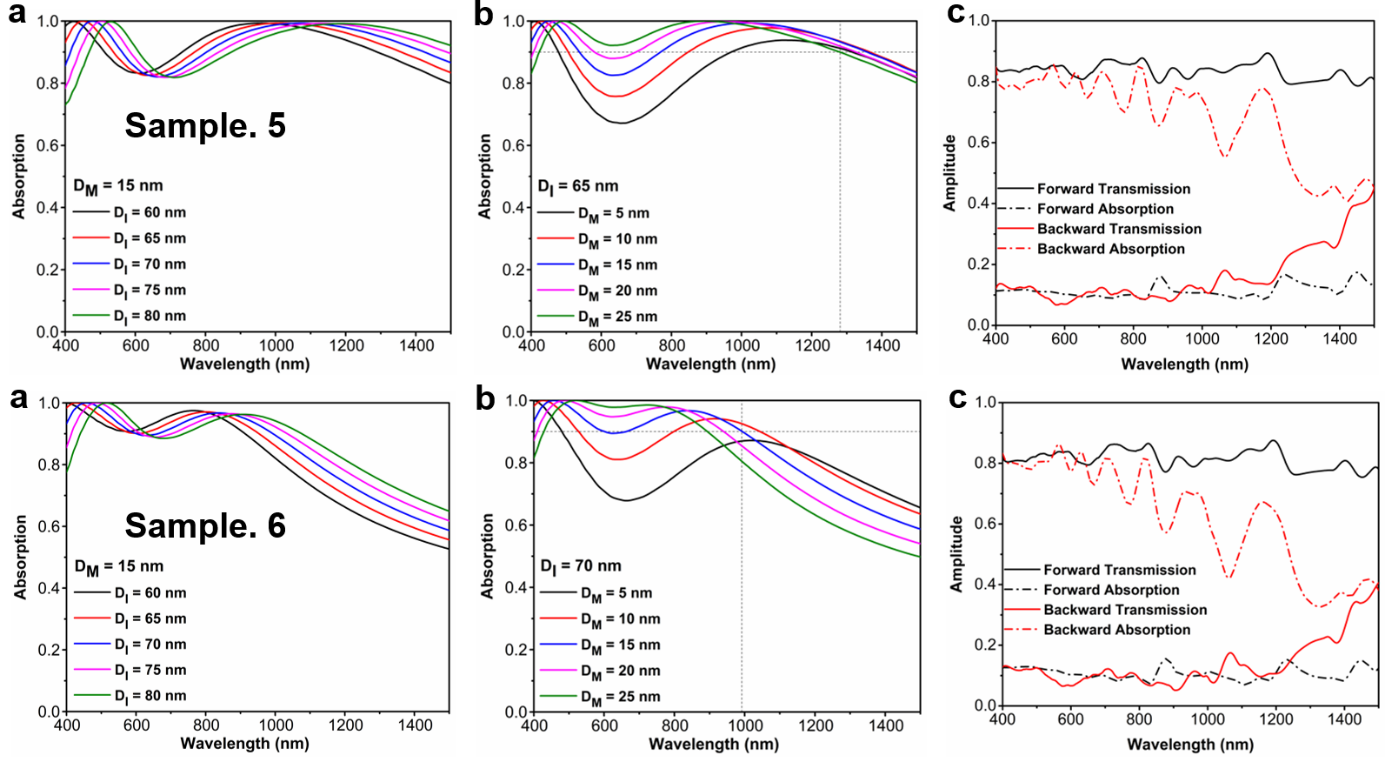

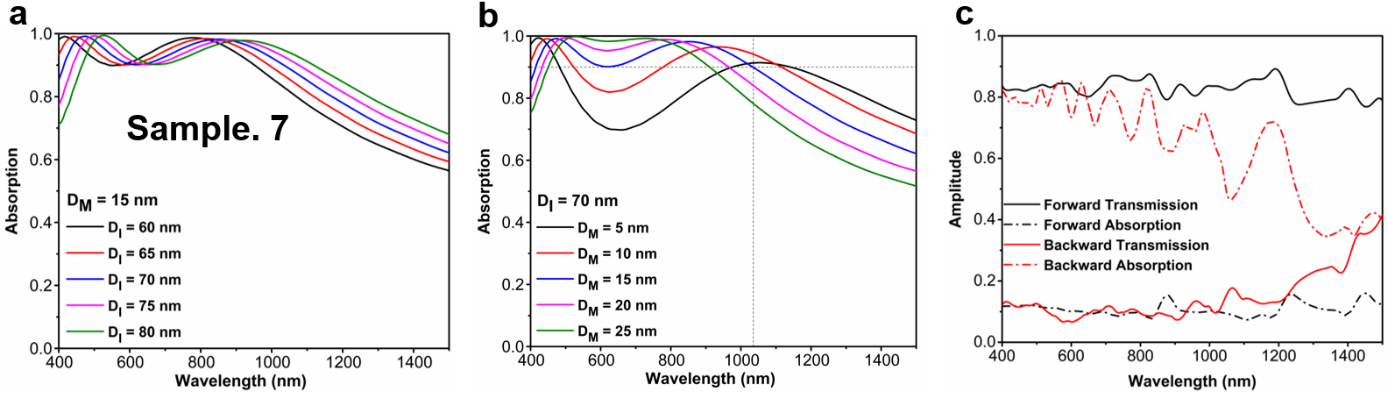


**Figure S1.** The absorption spectra as a function of different (a) insulator layer and (b) metal layer thicknesses. (c) The absorption ad transmission of the design for forward and backward light illumination.
